# Basal Activation of Type I Interferons (Alpha2 and Beta) and 2′5′OAS Genes: Insights into Differential Expression Profiles of Interferon System Components in Systemic Sclerosis

**DOI:** 10.1155/2011/275617

**Published:** 2011-11-01

**Authors:** Danilo Bretas de Oliveira, Gabriel Magno de Freitas Almeida, Antônio Carlos Martins Guedes, Flávia Patrícia Sena Teixeira Santos, Claudio Antônio Bonjardim, Paulo César Peregrino Ferreira, Erna Geessien Kroon

**Affiliations:** ^1^Laboratório de Vírus, Instituto de Ciências Biológicas, Universidade Federal de Minas Gerais, Avenue Antônio Carlos 6627, Pampulha 31270-901 Belo Horizonte, MG, Brazil; ^2^Hospital das Clínicas, Universidade Federal de Minas Gerais, Avenue Alfredo Balena 110, Santa Efigênia, 30130-100 Belo Horizonte, MG, Brazil

## Abstract

*Objective*. Systemic sclerosis (SSc) is a complex autoimmune disease in which interferons (IFNs) may play an essential role. We hypothesized that type I and III IFNs may be found in increased levels in patients and be responsible for SSc autoimmune status. *Methods*. Type I and III IFN and ISG basal expression profiles were measured by qPCR using RNA from PBMCs of patients and controls . *Results*. Type I IFNs are increased in SSc patients, while no induction of type III IFNs was detected. This induction cannot be related to IRF7, since no upregulation of this gene was seen on patients. Of the ISGs tested, 2′5′OAS levels were increased in patients, while 6–16 and MxA levels were not. *Conclusions*. While there is no indication of type III IFN induction, increased levels of type I IFNs may lead to abnormal regulation of ISGs that can be responsible for immune system alterations described for SSc.

## 1. Introduction

Systemic sclerosis (SSc) is a complex autoimmune disease of unknown aetiology characterized by excessive fibrosis of the skin and internal organs, presence of autoantibodies to nuclear antigens, and vascular damage [1–4]. Several genetic and environmental agents have been proposed to be responsible for causing SSc. Among these agents are infections, toxin exposure, single nucleotide polymorphisms (SNPs), and interferon treatment [5–7]. Regardless of the origin, the immune system on SSc patients shows evidence of homeostatic alterations, including increased levels of chemokines in blood serum and different populations of lymphocytes in peripheral blood mononuclear cells (PBMCs). In addition to these alterations, there is increased evidence that the interferon (IFN) system is modified in patients with SSc [8–15]. 

IFNs are immunomodulatory cytokines that act as an important link between the innate and adaptive immune system in vertebrates. IFNs bind to distinct cellular receptors, and their biological activities are mediated by the regulation of interferon stimulated genes (ISGs). IFNs can be divided into three types based on receptor binding and homology. Type I and type III IFNs are important regulators of innate immunity and are produced after stimulation of pattern recognition receptors in order to initiate and regulate the immune response. One of the main pathways leading to type I and III IFNs induction depends on the induction and activation of IRF7. Almost every cell type is able to produce these IFNs after stimulation, but its main producers are cells from the immune system such as plasmacytoid dentritic cells. Type I and III IFNs have redundant biological activities, even though they bind to different cellular receptors and have distinct structures. IFN gamma, the only known type II IFN, has distinct biological activities and is important for the regulation of adaptative immunity [16, 17]. The innate immune system and type I IFNs have been proposed as important factors in the initiation and maintenance of some autoimmune diseases, and the influence of type I IFNs in SSc has not been studied in detail [18–20]. Several activities of type I and type III IFNs are redundant, and until now there has been no description of type III IFNs participation in SSc or other autoimmune diseases. An SNP in one chain of the type III IFN receptor (IL10R2) has been associated with SSc, indicating that at least responsivity to these molecules may be important for the disease [21]. 

Previous studies have shown an increase of several ISGs in PBMCs of patients with autoimmune diseases such as systemic lupus erythematosus (SLE) and type I diabetes [10, 22]. Differential induction of ISGs in blood cells and fibroblasts from patients has already been described [19, 23–25]. When PBMC gene expression was compared by microarray between healthy donors and SSc patients, several ISGs were characterized as differentially induced. Some of these genes were also found in patients with SLE, which is an autoimmune disease marked by several alterations in the type I IFN system [4, 24]. Levels of 2′5′ oligoadenylate synthetase (2′5′OAS) and double-stranded RNA-activated protein kinase (PKR), two ISGs, are found at higher levels in fibroblasts from SSc patients when compared to controls [19]. To date, there is no direct evidence of abnormal induction of type I IFNs in SSc, even thought cells treated with sera from SSc patients produce more IFN alpha and other cytokines than cells treated with sera from controls [12]. These findings suggest that IFNs may also be important in SSc pathology. 

In this paper, we show that PBMCs from SSc patients basally expressed more type I IFNs (alpha and beta) than PBMCs from healthy donors. In addition, there was no detectable basal induction of type III IFNs in patients or healthy donors. When ISGs were measured, we observed increased 2′5′OAS basal levels, consistent with previous studies. These findings suggest that, similar to other autoimmune diseases, type I IFNs play an important role in SSc. 

## 2. Methods

### 2.1. Blood Donors

Ten patients fulfilling the American College of Rheumatology preliminary criteria for diagnosis of diffuse SSc and four healthy subjects were chosen as blood donors (Table 1). Before donating blood, each subject read and signed an informed consent previously approved by the Ethics Committee of the Universidade Federal de Minas Gerais, Brazil. 

### 2.2. Cells and PBMC Fractionation

Vero cells (African green monkey kidney cell line) were obtained from the ATCC and grown at 37°C in Dulbecco's modified Eagle's medium (DMEM) supplemented with 2 mM glutamine, 5% fetal calf serum (Cultilab, Campinas, SP, Brazil), and antibiotics. PBMCs from healthy donors or SSc patients were purified using the Ficoll-Hypaque purification technique [26]. Briefly, fresh blood collected in vacuum tubes containing heparin was diluted in an equal volume of 1X phosphate buffered saline (PBS). Twenty milliliters of the diluted blood were carefully added over 10 mL of Ficoll and centrifuged at 400 × g for 30 min. The layer containing PBMCs was collected and washed once in 1X PBS, and the cells were counted. One million cells were pelleted and used for RNA extraction.

### 2.3. RNA Extraction, DNase Treatment, Reverse Transcription and Quantitative PCR (qPCR)

Total cellular RNA was extracted using the RNeasy mini kit (QIAGEN). After the extraction, one microgram of RNA was treated with 1 U of DNase I enzyme (BIOLABS), and after treatment the RNA was used as template in reverse transcriptions carried out using MMLV reverse transcriptase (PROMEGA). These steps were performed as described by the manufacturers. Real time PCRs were performed in a Step-One Real-Time PCR machine (Applied Biosystem) using the relative quantification methodology. The results were analyzed using StepOne Software v2.2, and all data were expressed as a ratio relative to the beta-actin level. PCR primers used for human genes are listed below: IFN alpha2 forward 5′-TTGACCTTTGCTTTACTGGT-3 and reverse 5′-CACAAGGGCTGTATTTCTTC-3′. IFN beta forward 5′-CCTGTGGCAATTGAATGGGAGGC-3′ and reverse 5′-CAGGTAGATGGTATAGCGTGG-3′. IFN lambda1 forward 5′-CTTCCAAGCCCACCCCAACT-3′ and reverse 5′-GGCCTCCAGGACCTTCAGC-3′. IFN lambda2/3 forward 5′-TTTAAGAGGGCCAAAGATGC-3′ and reverse 5′-TGGGCTGAGGCTGGATACAG-3′. IRF-7 forward 5′-CAAGTGCAAGGTGTACTGG-3′ and reverse 5′-CAGGTAGATGGTATAGCGTGG-3. 2′5′OAS forward 5′-AACTGCTTCCGACAATCAAC-3′ and reverse 5′-CCTCCTTCTCCCTCCAAAA-3′. MxA forward 5′-ATCCTGGGATTTTGGGGCTT-3′ and reverse 5′-CCGCTTGTCGCTGGTGTCG-3′. 6–16 forward 5′-CATGCGGCAGAAGGCGGTAT-3′ and reverse 5′-CGACGGCCATGAAGGTCAGG-3′. Beta-actin forward 5′-CCAACCGCGAGAAGATGA-3′ and reverse 5′-CCAGAGGCGTACAGGGATAG-3′.

### 2.4. IFN Titration

After cell fractionation by the Ficoll-Hypaque technique, one milliliter of sera was collected from each donor and frozen at −70°C until use. At the time of titration, each serum sample was serially diluted from 1 : 2 to 1 : 4096 and used to treat 96-well plates of Vero cells with 90% confluency. Eighteen hours after treatment, the medium was discarded and the cells were infected with 10^4^ TCID50/mL of *Encephalomyocarditis virus* (EMCV). The infection was monitored for 48 hours, at which time the plates were fixed with 3.7% formaldehyde before being stained with 1% crystal violet (adapted from [27]). Alongside the samples, the following controls were used: 600 U/mL of recombinant human IFN alpha 2a (Roche) and a negative serum sample to which 600 U/mL of recombinant human IFN alpha 2a (Roche) was added.

### 2.5. Statistical Analysis

Student's *t*-test and nonparametric Mann-Whitney test were used to analyze the results. Differences of *P* < 0.05 were considered to be statistically significant. Analyses were made using the GraphPad Software (USA)

## 3. Results

**Figure 1:**
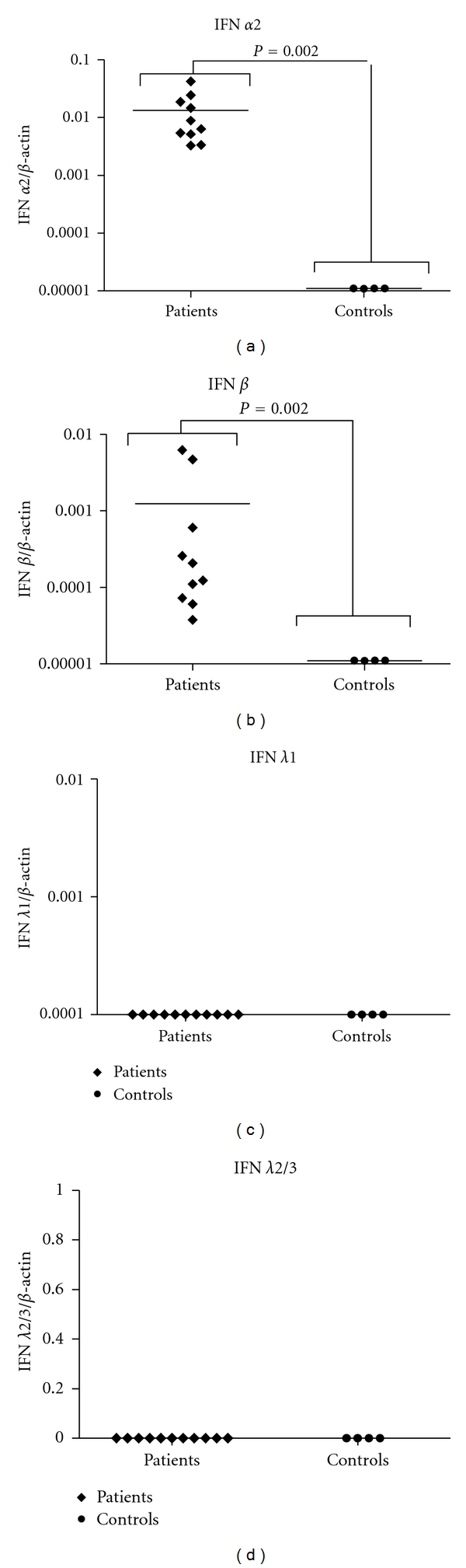
Type I and type III IFN mRNA basal levels in PBMCs from healthy donors and SSc patients. PBMCs from healthy donors and SSc patients were purified, and total RNA extraction was performed. The RNA obtained was used as template in reverse transcription reactions, and the resulting cDNA was used in real-time PCRs to measure IFN alpha (a), IFN beta (b), IFN lambda1 (c), and IFN lambda 2/3 (d).

The expression profile of type I and type III IFNs was measured by qPCR using RNA from PBMCs freshly collected from SSc patients or controls. We observed that the basal levels of IFN alpha2 and IFN beta were increased in patients while there was little or no expression of these genes on healthy donors (Figures 1(a) and 1(b)). When type III IFNs were measured, we observed that the basal level of IFN lambda1 and lambda2/3 was the same in both groups (Figures 1(c) and 1(d)). These results were obtained from cells without any treatment or culture, indicating that these genes might be normally induced in patients. We also attempted to detect IFN proteins in sera from patients and controls through a biological assay commonly used for IFN titration. However we were unable to detect any activity higher than the detection limit of the assay (20 IU/mL). Our controls worked perfectly, indicating that the negative results obtained were not due to sera toxicity or experimental artifacts (data not shown). 

**Figure 2:**
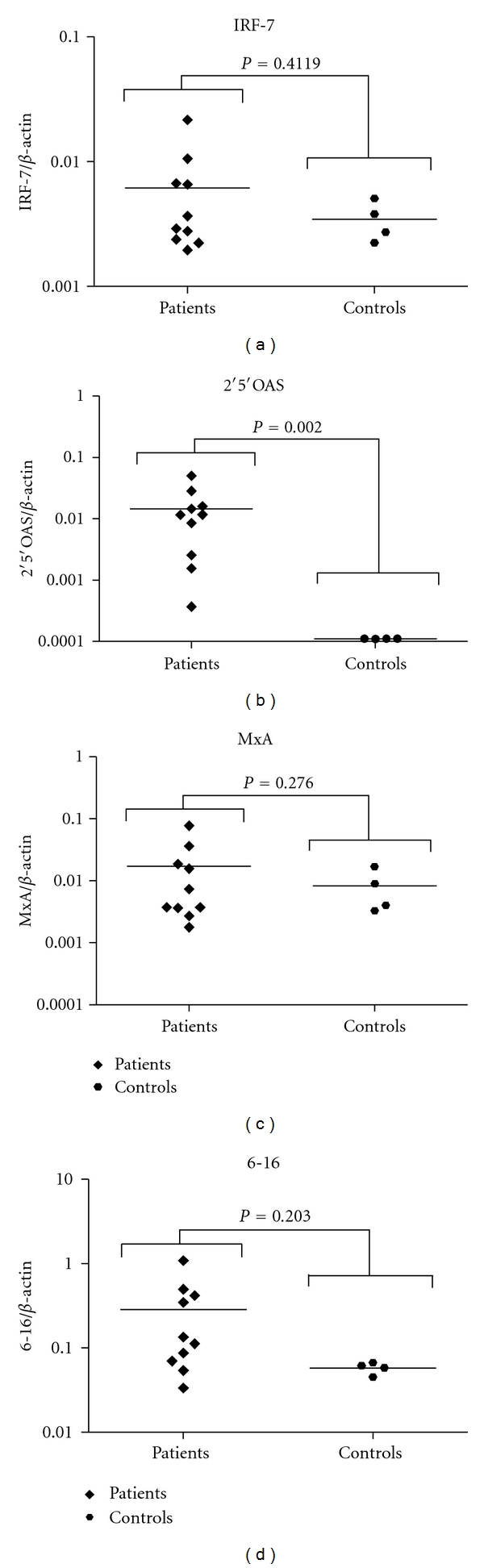
Basal levels of IRF-7 and ISGs in PBMCs from healthy donors and SSc patients. PBMCs from healthy donors and SSc patients were purified, and total RNA extraction was performed. The RNA obtained was used as template in reverse transcription reactions, and the resulting cDNA was used in real-time PCRs to measure IRF-7 (a), 2′5′OAS (b), 6–16 (c), and MxA (d) levels.

In addition to IFN basal levels, we also measured IRF7, 2′5′OAS, MxA, and 6–16 basal levels in PBMCs from patients and controls. IRF7 is an important factor for type I and type III IFN induction, whereas the other genes are ISGs that are commonly induced by type I and type III IFNs. There was no statistically significant difference in the levels of IRF7 (Figure 2(a)) between patients and controls. Similarly, there was no difference between levels of MxA (Figure 2(c)) and 6–16 (Figure 2(d)). However a difference on 2′5′OAS levels was detected, and this gene basal expression level was increased in patients but not in controls (Figure 2(b)). 

## 4. Conclusions

IFNs are cytokines with antiviral, antiproliferative, and immunomodulatory activities. Type I IFNs have been implicated in the pathogenesis of several known autoimmune diseases. However, the influence of type III IFNs in these diseases is not yet known, despite the similarities in the biological activities of these IFN types. SSc is an autoimmune disease with unknown etiology in which the immune system homeostasis is severely compromised. To date there has been little information about the influence of IFNs in SSc patients in the literature and no direct evidence of type I or type III IFN basal induction in patients.

In this paper, we show that some components of the IFN system can be found at higher levels in freshly purified PBMCs from SSc patients, compared with cells from controls. Increased basal expression of IFN alpha2 and IFN beta (type I IFNs) was detected in SSc patients, while there was no difference between the basal levels of IFN lambda1 and IFN lambda2/3 (type III IFNs) in patients and controls (Figure 1). These results show that, at least for mRNA levels, there is a higher expression of type I IFNs by PBMCs from SSc patients than from controls. This abnormal expression, especially in the case of IFN alpha2, is consistent with the status of autoimmunity in several other diseases and can have a major role in initiating and maintaining the disease. High levels of IFN beta can be even more dramatic for an autoimmune status, since this IFN has similar biological activities to IFNs alpha but is even more potent [28]. We also tried to titrate IFN protein levels on sera obtained from patients and controls, but the levels were below our limit of detection (20 IU/mL). Considering that this titration method is able to detect any mixture of IFNs capable of inducing antiviral activity in host cells, we can hypothesize that even though IFN levels are increased on SSc, they are not found in high levels. Methodologies with greater sensitivity must be used in order to detect and measure type I IFN proteins in SSc patients sera.

Expression levels of IRF7, an inducible transcription factor responsible for type I and type III IFN gene induction [29], were not altered between patients and controls (Figure 2(a)). This finding leads us to conclude that IFN alpha2 and beta are being induced by another stimulus in the disease, which is specific to their induction and is not common to type III IFNs induction. 2′5′OAS basal expression levels were higher in patients than in controls (Figure 2(b)). This gene is also found at higher levels in SLE patients [30]. Differential induction of 2′5′OAS is consistent with a previous study that used fibroblasts from SSc patients, in which higher basal levels of 2′5′OAS were detected when compared to control fibroblasts [19]. It also indicates that there is, at least to some degree, some similarity between alterations in the skin and blood cells of SSc patients. Other measured ISGs, 6–16, and MxA were not differentially expressed between patients and controls (Figures 2(c) and 2(d)). The factors leading to this differential induction on ISGs are unknown, but could be due to cell exposure to various IFN subtypes abnormally expressed during the disease. The capacity of SSc sera to induce cytokines in treated cells [31] supports this observation, since this activation probably occurs due to cytokines and other molecules in the plasma. 

Based on these findings, we hypothesize that the IFN system is modified in SSc. By still unknown mechanisms, at least IFNs alpha2 and IFN beta are induced in patients by a pathway that does not induce type III IFNs. Type I IFNs produced by patients' cells circulate and through activation of certain subsets of ISGs maintain the autoimmune status of SSc. These molecules are important links between innate and adaptative immune responses and can, among other actions, activate immune cells to improve autoantigens detection and autoantibodies production [32]. It has been already described that IFN alpha2 can induce TLR3 activation in SSc fibroblasts in culture [33]. This activation can also be happening *in vivo* by type I IFNs naturally produced by PBMCs, corroborating our hypothesis. IFN beta activation is a novel finding on the disease and can be of great importance to SSc altered immune homeostasis. Even though there is some redundancy between the activity of type I and type III IFNs, we could not link type III IFNs to SSc, at least when PBMCs are involved. Future experiments with higher sample size and using distinct cell populations isolated from total PBMCs are essential to further investigate these results. 

These findings are of great importance to our understanding of the pathogenesis and pathology of SSc. Increased basal levels of type I IFNs can lead to abnormal activation of ISGs and undesirable side effects on the patients, even without interference by type III IFNs. The differential expression profile of ISGs detected in PBMCs is consistent with the profile found previously in fibroblasts [19] and could be a signature related to the disease. These unique modifications in the IFN response may be considered as candidates for SSc biomarkers and can be considered for further studies as diagnostic tools for SSc. 

## Figures and Tables

**Table 1:** Information about the subjects that consented to take part in this research.

	Sex	Age (years)	Disease time (years)^1^	Treatment	ANA titer
**P1**	F	31	1	Prednisone	>1/320
**P2**	F	54	5	Methotrexate Prednisone	<1/10240
**P3**	M	35	5	Methotrexate Prednisone	>1/5120
**P4**	M	29	1	Prednisone	>1/640
**P5**	M	40	7	—	<1/10240
**P6**	F	30	2	—	>1/320
**P7**	F	50	5	—	>1/80
**P8**	M	56	6	—	<1/10240
**P9**	F	58	11	Methotrexate Prednisone	>1/80
**P10**	F	70	20	—	>1/640
**C1**	M	39	—	—	Not tested
**C2**	F	32	—	—	Not tested
**C3**	M	25	—	—	Not tested
**C4**	F	24	—	—	Not tested

C: control; P: patient; M: male; F: female; —: not treated.

^1^Dated from the onset of the first non-Raynaud's symptom.
